# A Novel Method of Combining Blood Oxygenation and Blood Flow Sensitive Magnetic Resonance Imaging Techniques to Measure the Cerebral Blood Flow and Oxygen Metabolism Responses to an Unknown Neural Stimulus

**DOI:** 10.1371/journal.pone.0054816

**Published:** 2013-01-31

**Authors:** Aaron B. Simon, Valerie E. M. Griffeth, Eric C. Wong, Richard B. Buxton

**Affiliations:** 1 Department of Bioengineering and Medical Scientist Training Program, University of California San Diego, La Jolla, California, United States of America; 2 Center for Functional Magnetic Resonance Imaging, Department of Radiology, University of California San Diego, La Jolla, California, United States of America; 3 Kavli Institute for Brain and Mind, University of California San Diego, La Jolla, California, United States of America; University of Medicine & Dentistry of NJ - New Jersey Medical School, United States of America

## Abstract

Simultaneous implementation of magnetic resonance imaging methods for Arterial Spin Labeling (ASL) and Blood Oxygenation Level Dependent (BOLD) imaging makes it possible to quantitatively measure the changes in cerebral blood flow (CBF) and cerebral oxygen metabolism (CMRO_2_) that occur in response to neural stimuli. To date, however, the range of neural stimuli amenable to quantitative analysis is limited to those that may be presented in a simple block or event related design such that measurements may be repeated and averaged to improve precision. Here we examined the feasibility of using the relationship between cerebral blood flow and the BOLD signal to improve dynamic estimates of blood flow fluctuations as well as to estimate metabolic-hemodynamic coupling under conditions where a stimulus pattern is unknown. We found that by combining the information contained in simultaneously acquired BOLD and ASL signals through a method we term BOLD Constrained Perfusion (BCP) estimation, we could significantly improve the precision of our estimates of the hemodynamic response to a visual stimulus and, under the conditions of a calibrated BOLD experiment, accurately determine the ratio of the oxygen metabolic response to the hemodynamic response. Importantly we were able to accomplish this without utilizing *a priori* knowledge of the temporal nature of the neural stimulus, suggesting that BOLD Constrained Perfusion estimation may make it feasible to quantitatively study the cerebral metabolic and hemodynamic responses to more natural stimuli that cannot be easily repeated or averaged.

## Introduction

Functional hyperemia is a phenomenon by which blood flow to a volume of brain tissue increases rapidly and dramatically in response to a local increase in neural activity. Though still poorly understood, functional hyperemia is thought to play an important role in the maintenance of homeostasis in the brain, and its dysfunction has been postulated to play a role in the etiologies of several cerebral vascular and neurodegenerative diseases [1].

Functional Magnetic Resonance Imaging (fMRI) has become a popular method of studying functional hyperemia in humans, both because it is non-invasive and because it is capable of imaging large volumes of tissue with good spatial and temporal resolution. The most commonly used fMRI technique today is blood oxygenation level dependent (BOLD) imaging. Contrast in BOLD imaging is derived from the paramagnetic properties of deoxygenated hemoglobin, which increases the transverse relaxation rate of the MR signal [2]. In general, functional hyperemia leads to a local decrease in the fraction of oxygen extracted from capillaries, increasing the oxygenation of hemoglobin in downstream venules [3] and producing a robust increase in the BOLD signal. BOLD imaging is highly sensitive to fluctuations in blood oxygenation and is thus often used to localize regions of the brain where blood oxygen saturation changes in response to neural activity. However, BOLD imaging is limited in two ways. First, it cannot be interpreted in a quantitative physiological sense, as both the rate of delivery and rate of consumption of oxygen affect the magnitude of the BOLD signal and cannot be disentangled by BOLD imaging alone [4]. Second, the BOLD signal is a change between two acutely defined states, and so is not directly sensitive to chronic physiological changes that would affect the baseline state.

Arterial spin labeling (ASL), an MR imaging technique that creates contrast by magnetically labeling arterial blood as it enters the cerebrovasculature, is a more direct method of imaging functional hyperemia and in principle overcomes the two limitations of BOLD imaging noted above [5], [6]. Like BOLD imaging, ASL is non-invasive and sensitive to the fluctuations in local blood flow that accompany neural activity. However, unlike BOLD imaging, ASL can provide quantitative information about the local perfusion in absolute physiological units, including both the baseline and activated states, making it a potentially highly useful tool for understanding brain function and cerebrovascular physiology in health and disease [6], [7]. In addition, as a component of a multi-modal imaging approach including simultaneous BOLD imaging, ASL may be used to disentangle competing neuronal and vascular contributions to the BOLD signal, allowing quantitative measurement of CMRO_2_ fluctuations [8], [9]. However, ASL suffers from several limitations of its own. Amongst the greatest limitations of this technique is the intrinsically low signal-to-noise ratio of the ASL signal, which is largely due to the small amount of labeled arterial blood that can be delivered during the longitudinal relaxation time of the blood. To compensate, quantitative measurements of CBF using ASL are often made with lower spatial and temporal resolution than standard BOLD-fMRI studies, and are primarily used to measure baseline blood flow. Dynamic measurements of blood flow typically require significant trial averaging over repeated stimuli and spatial averaging over a selected region of interest [4], [10]–[12].

While such studies provide useful insights into differences in cerebral perfusion and metabolic requirements between nominal states of “control” and “activity”, the methods they employ can say little about the role of functional hyperemia in everyday neural processing. Current methods of quantitatively estimating CBF and CMRO_2_ fluctuations associated with more natural neural tasks, which could include watching a film, listening to music, or simply lying quietly in the scanner, are inadequate in large part because the underlying stimulus driving hemodynamic and metabolic changes cannot be defined, making it difficult to identify and average measurements corresponding to the same physiological state. Here we examined the feasibility of estimating fluctuations in CBF and CMRO_2_ without *a priori* knowledge of the temporal pattern of neural activity by combining the CBF information contained in simultaneously acquired BOLD and ASL measurements. We hypothesized that because the BOLD signal is strongly driven by CBF, simultaneous measurement of ASL and BOLD fluctuations via a combined BOLD-ASL imaging experiment could be used to model an improved estimate of the “true” CBF signal even in the presence of significant noise in both the BOLD and ASL measurements. Further, we hypothesized that under the conditions of a calibrated BOLD experiment, information about fluctuations in CMRO_2_ could also be extracted from the information contained in the combined BOLD-ASL data. Importantly, we hypothesized that correction of the CBF signal could be accomplished without explicit, *a priori* knowledge of the stimulus presented, opening up a path towards the quantitative study of how cerebral blood flow is modulated to meet the metabolic demands of the neural processing that occurs in response to natural stimuli or at rest. We call this combination of BOLD and ASL image data BOLD-Constrained Perfusion (BCP) estimation to emphasize that our criteria for distinguishing CBF signal fluctuations from noise is that they be simultaneously reflected in the BOLD signal.

**Figure 1:**
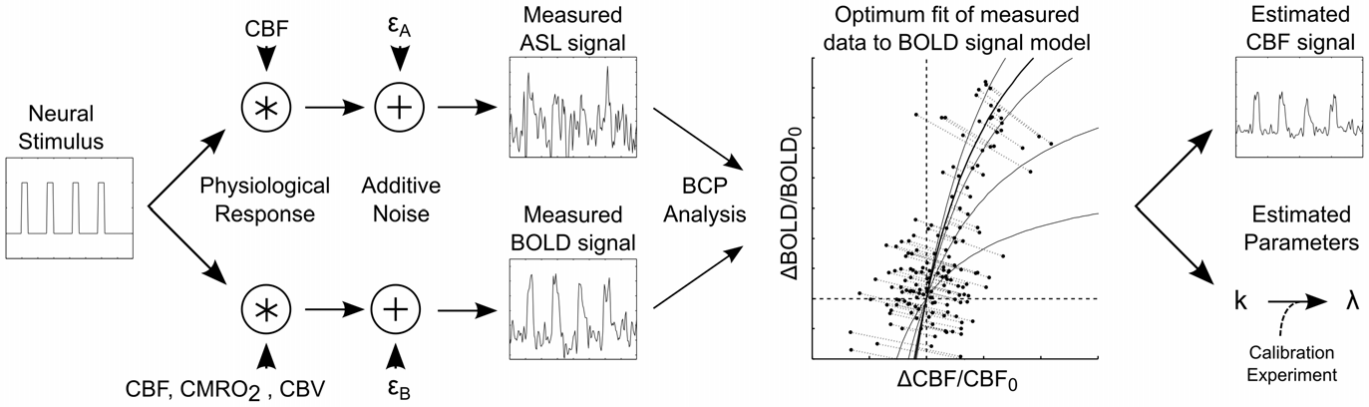
Schematic of the BOLD-constrained Perfusion (BCP) estimation process. When a cognitive task is presented to a subject, induced neural activity evokes both a hemodynamic and a metabolic response. ASL imaging captures principally the evoked changes in CBF while BOLD imaging is sensitive to changes in CBF, CMRO_2_, and CBV. In addition, both imaging modalities are sensitive to noise of both physiological and mechanical origin (ε_A_ and ε_B_, respectively). The BCP analysis approach is to combine information about CBF fluctuations present in both the BOLD and ASL signals to improve the estimate of dynamic CBF fluctuations. This is accomplished by fitting the measured data to a cost function (Equation 4 in text) that treats the measured time series as noisy representations of two signals that are linked by a simple mathematical model. The output of this process is an improved dynamic estimate of CBF fluctuations. Under the conditions of a calibrated BOLD experiment, an additional estimated parameter of the mathematical model (*k*) may also provide information about the coupling of CMRO_2_ and CBF fluctuations (λ). *ASL: Arterial Spin Labeling. CBF: Cerebral Blood Flow. CMRO_2_: Cerebral Metabolic Rate of Oxygen Metabolism. CBV: Cerebral Blood Volume.*

The outline of this paper is as follows. We begin with a short theoretical discussion of our motivation for pursuing the BCP estimation approach, which we illustrate schematically in Figure 1. We then present empirical results that demonstrate that the BCP approach can both increase the precision of dynamic CBF estimates and, under the conditions of a calibrated experiment, provide information about the coupling of CBF and CMRO_2_ without *a priori* knowledge of the stimulus driving neural activity. In order to be able to validate the results of the BCP analysis, we analyzed data that were previously acquired and reported as a calibrated BOLD study [8], using a stimulus with well-understood temporal characteristics: a simple block-design visual task. Our rationale for choosing such a simple stimulus was two-fold. First, to determine whether the influence of noise on the estimated dynamic CBF time series was decreased by BCP analysis, we needed to be able to predict with some confidence what the CBF time series should be in the absence of noise. Second, in order to be able to verify that we could accurately estimate the coupling of CBF and CMRO_2_ we needed to choose an experimental design for which traditional calibrated BOLD analysis could also be performed.

## Theory

### Signal and Noise in Simultaneous BOLD-ASL Imaging

In a dual-echo, simultaneous BOLD-ASL acquisition scheme, “tag” images, in which the magnetization of inflowing arterial blood is inverted, and “control” images, in which the magnetization of arterial blood is not inverted, are acquired in an interleaved fashion, typically with an echo-planar or spiral gradient recalled echo (GRE) readout. The echo time (TE) of the first echo is chosen to be as short as possible in order to minimize sensitivity to fluctuations in 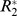 decay, while the second echo is chosen to have a longer TE, so as to maximize BOLD sensitivity. From the measured time series two new time series are constructed by surround subtraction and surround addition, in which the voxel signal at one time point is appropriately combined (subtracted or added) with the average value of the preceding and following time points. Surround subtraction of sequential images acquired at the first echo time produces the ASL time series of images, in which the intensity of each voxel is weighted by the local rate of cerebral blood flow. Surround addition of sequential second echo images produces the BOLD time series of images, with little CBF weighting but considerable BOLD sensitivity [13].

However, in addition to CBF and blood oxygenation, the instantaneous magnitudes of the surround subtraction and surround addition signals, respectively, are sensitive to several sources of noise. This noise may be attributable to the scanner itself, to subject motion and cardiac pulsatility, or to instabilities in the magnetic field due to changes in the size of the thoracic cavity associated with the breathing cycle. Several methods have been developed for identifying and removing the signal contributions from some of these noise sources, in particular, subject motion, cardiac and respiratory activity, and scanner drifts [14]–[19]. Often, one or more of these methods is used to reduce the noise in the BOLD and ASL signals before quantitative analysis is performed. However, in general, none of these techniques can perfectly remove all sources of nuisance signal and no technique can remove the random thermal noise inherent in every signal. Thus we must think of our measured BOLD and ASL signals, even after correction for known sources of noise, as discrete time signals that are combinations of both “real” signal fluctuations (that are of interest to us) and noise [13]. We can express this very generically as
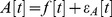
(1)

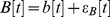
(2)where 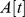 and 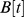 are the measured ASL and BOLD signals, respectively, 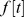 represents a low-pass filtered representation of the CBF at sample *t* scaled by a constant related to imaging parameters and experimental conditions, 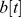 represents a low-pass filtered representation of the instantaneous BOLD signal, and 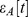 and 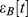 capture the contributions of random thermal noise and any physiological sources of noise in the ASL and BOLD signals, that are not completely removed by the methods cited above. The precision of the CBF and BOLD estimates at each time point is then determined by the variances of 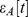 and 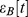, which may still be significant, especially for the ASL signal.

### Relating BOLD Fluctuations to Changes in Cerebral Blood Flow

Both 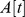 and 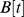 are driven by the underlying CBF fluctuations. The former is a direct but noisy reflection of CBF, and the latter is a more sensitive measurement but related to CBF in a nonlinear way. The central idea of BOLD-constrained perfusion (BCP) is to use both signals to make a better estimate of the underlying CBF fluctuations. To utilize the BOLD signal in this way requires a mathematical model that links changes in cerebral blood flow to changes in the BOLD signal. Recently our group developed a detailed numerical model of the BOLD response as a function of changes in CMRO_2_, CBF, and cerebral blood volume (CBV). The model also includes a number of baseline physiological parameters (microvascular hematocrit, venous and capillary blood volume, baseline oxygen extraction fraction (OEF), etc.) that modulate the magnitude of the BOLD response [20]. Although the detailed model is not in a tractable form for the current application, it nevertheless provides a useful framework for testing the accuracy of much simpler, closed-form models. Recently we used this approach to develop a relatively simple model and test its accuracy through many simulations with the detailed model for different values of the unknown physiological parameters [14]. The form of the model is:
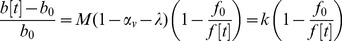
(3)


In this equation, the parameter *M* is a scaling factor that absorbs many of the physiological factors that simply scale the BOLD response and depends on the amount of deoxyhemoglobin in the baseline state as well as parameters of the image acquisition (echo time and field strength). We have used the symbol *M* for this scaling factor in analogy with the Davis model [21], but it should be noted that analyzing data to determine a value of *M* will yield a different numerical value using Equation 3 than using the original Davis model because of the different mathematical form. The factor *α_v_* is the exponent of a power law relationship between the venous CBV change and the CBF change. The parameter λ is the ratio of the fractional change in CMRO_2_ to the fractional change in CBF (e.g., a 20% change in CMRO_2_ with a 40% change in CBF would correspond to λ = 0.5). Finally, *f_0_* and *b_0_* represent the magnitude of the CBF and BOLD signal in the baseline state.

We refer to this model as a *heuristic* model because it clearly shows the basic anatomy of the BOLD response: it is driven by the CBF change, but strongly modulated by the baseline state (*M*), the venous CBV change (*α_v_*), and the CMRO_2_/CBF coupling ratio (λ). In addition, though, our comparison tests with the detailed model have shown that the heuristic model is reasonably accurate as well. Previously, the most commonly used closed form model for the BOLD response was the Davis model, and our comparison tests have shown that the accuracy of these two simpler models is similar. However, the particular advantage of the heuristic model is that all of the unknown parameters that modulate the BOLD response can be combined into a single factor, *k*, scaling a simple nonlinear function of the CBF change. This means that our model connecting 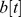 to the underlying CBF fluctuation requires only a single parameter to be determined. (In principle, the BCP approach can be applied using any model that connects the BOLD response to the CBF change, and the Supporting Section and shows a similar analysis based on the Davis model. See the Document S1, Figure S1, and Figure S2 for this analysis).

### BOLD Constrained Perfusion Estimation

We now propose that the precision of an estimate of the instantaneous CBF, 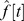 may be improved by assuming that the expected values of the measured ASL and BOLD signals, 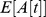 and 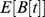 are the true underlying CBF and BOLD signals, 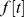 and 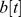, and that the unknown parameter *k* of our BOLD model has a constant value over a window of interest *T* samples in length. The values of 
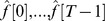
 and 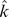 can then be estimated by minimizing the cost function
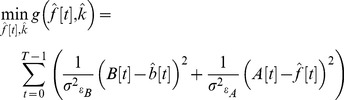
(4)under the constraint that 
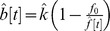
 (Equation 3) at every time point in the window. In essence, what we are doing here is finding the values of 
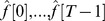
 and 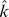 that best fit the measured ASL and BOLD signals to Equation 3 given the assumption that there is noise in both (Figure 1). Note that this is quite different from performing a simple non-linear regression of the two signals based on Equation 3, which would implicitly assume that only one of the signals contained noise and that the other was noise-free. In the cost function represented by Equation 4, the parameters 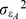 and 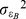 are weighting parameters that reflect the fact that both measured signals contain noise and account for the possibility that the noise variance in 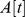 may be different from that of 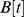. In this work we have estimated these parameters by measuring the variances of the BOLD and ASL signals in voxels containing cerebral spinal fluid (CSF), which should have no CBF-related ASL or BOLD signal fluctuations [17].

### Estimating CMRO_2_-CBF Coupling for Calibrated Experiments

As stated above, the output of the minimization of Equation 4 is a time series of CBF estimates, 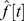, which we hypothesize will more precisely approximate the time course of true underlying CBF fluctuations than the time series of ASL measurements 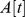. In addition, the minimization of Equation 4 yields an estimate of the parameter *k*, which links BOLD fluctuations to underlying changes in CBF. Alone, the value of 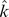 yields very little information of physiological interest. This is because the value of *k* depends on three parameters, α_v_, λ, and *M*. However, if the values of *M* and α_v_ are obtained by other means, then an estimate of *k* becomes equivalent to an estimate of λ. This is of great physiological interest as it represents the ratio of CMRO_2_ changes to CBF changes throughout the analysis window. For a typical calibrated-BOLD experiment, the value of α_v_ is assumed based on literature values. Though there is still some disagreement about the appropriate value to assume for α_v_, the most recent estimates suggest that it is approximately 0.2 [22]. Because of the dependence of *M* on the baseline state, in most cases it must be measured rather than assumed. The most common method of estimating *M* is through a separate calibration experiment during which simultaneous BOLD and ASL images are acquired while the subject breaths CO_2_ enriched air [23]. The underlying assumption of this experiment is that breathing CO_2_ increases blood flow without affecting oxygen metabolism (λ = 0), allowing one to calculate *M* based on an assumed value of α_v_. Of course, the accuracy of the value of λ estimated by this approach will depend on the accuracy of the values obtained for α_v_ and *M*. However, under conditions where these values may be obtained, BCP analysis may yield an estimate of CMRO_2_-CBF coupling in addition to CBF fluctuations.

### Potential Sources of Bias in BCP estimates

The BCP approach outlined here relies implicitly on several assumptions about the nature of both the underlying physiology of functional hyperemia and the characteristics of the BOLD and ASL signals that could potentially bias BCP estimates of 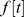 and λ. First, the BCP approach assumes that the CMRO_2_-CBF coupling ratio (λ) varies slowly enough in time that it can be considered constant over a window of several or even many time points. If in reality λ varies significantly over a period of time shorter than the window, the BCP estimates of both λ and 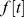 may become less accurate. Related to this is the assumption that the dynamics of the CBF, CBV and CMRO_2_ responses to neural stimuli are tightly coupled, at least as resolvable at the sampling rate of an ASL experiment. In reality the coupling of these processes may not be strictly tight, as analysis of transient features of the BOLD response have led several investigators to conclude [24]–[27]. Dynamic mismatch of these processes would effectively produce transient fluctuations in the values of λ or α_v_ in Equation 3, which again is not currently accounted for in the BCP approach. Finally, the BCP approach is based on the idea that CBF fluctuations may be distinguished from noise by the correlated fluctuations they produce in the ASL and BOLD signals. This implicitly assumes that noise in the ASL and BOLD signals is not correlated in a significant way. If correlated noise in the ASL and BOLD signals is consistent with a similar estimate of *k* as the physiological fluctuations in CBF and CMRO_2_, the correlated fluctuations will add noise to the BCP estimation, reducing the ability of BCP estimation to improve the precision of 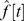. If the correlated noise leads to a shift in the line defining the BOLD-ASL relationship it will likely reduce the accuracy of both 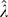 and 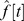 as well as their precisions.

## Methods

### Ethics Statement

This study was approved by the institutional review board at the University of California San Diego, and written informed consent was obtained from all participants.

### Imaging

For the empirical component of this work, we reanalyzed the raw data from a previously published calibrated-BOLD study [8]. Briefly, the study was conducted on 10 healthy adults (mean age 33+/−7 years). Simultaneous BOLD and CBF images were acquired on a GE Excite 3T scanner with a dual-echo arterial spin labeling (ASL) PICORE QUIPSS II sequence [28] with a spiral readout. ASL sequence parameters were six 5-mm slices aligned with the calcarine sulcus, TR 2.5 s, TI1/TI2 600/1500 ms, TE1 2.9 ms, TE2 24 ms, 90° flip angle, FOV 240 mm, matrix 64 × 64. Functional imaging consisted of two scans during which subjects performed a visual task and two calibration scans during which subjects breathed a gas mixture containing 5% CO_2_. Each visual task began with 60 seconds of rest followed by four cycles of 20 seconds of stimulus, 60 seconds of rest, and ended with a final 30 seconds of rest. The stimulus consisted of a black and white checkerboard flickering at 8 Hz while numbers appeared in the center of the checkerboard. Throughout scanning, cardiac pulse and respiratory effort data were monitored using a pulse oximeter (InVivo) and a respiratory effort transducer (BIOPAC), respectively. A high-resolution anatomical image was also acquired at the start of each session, using a magnetization prepared 3D fast spoiled gradient acquisition in the steady-state (FSPGR) sequence (172 sagittal slices, 1-mm slice thickness, TI 450 ms, TR 7.9 ms, TE 3.1 ms, 12° flip angle, FOV 25 cm, matrix 256×256).

### Preprocessing

The first four images of each ASL scan were excluded from data analysis to allow the MRI signal to reach steady state. All functional runs were motion corrected and registered to the first functional run using AFNI software [29]. The first echo data was used for the analysis of CBF activity, and the second echo data for the analysis of BOLD activity. To generate a perfusion-weighted signal from the raw ASL images at each time point during the functional scans, the image intensity corresponding to a “tag” image was subtracted voxel-wise from the average intensity of the surrounding two “control” images. Similarly, at each “control” time point, the image intensity was added voxel-wise to the negative average of the surrounding two “tag” images. BOLD-weighted images were obtained by adding the image intensity at each time point (tag or control) to the average of the intensities in the two surrounding time points [13].

### ROI Selection

ROI selection was performed on the data from the first visual task using a general linear model (GLM) approach for the analysis of ASL data [8], [16]. A stimulus-related regressor in the GLM was obtained by convolving the block design stimulus pattern with a gamma density function [30]. The measured cardiac and respiratory data were included in the GLM as regressors to account for the modulation of the ASL signal caused by physiological fluctuations [15], [16] as were regressors related to variations in heart rate and respiratory volume [19]. A constant and a linear term were also included as nuisance regressors to account for scanner drift. Voxels exhibiting CBF or BOLD activation were detected after correcting for multiple comparisons using AFNI AlphaSim [29], [31] and setting an overall significance threshold of p = 0.05 for CBF and p = 0.01 for BOLD given a minimum cluster size of four voxels. For each subject, an active region of interest (ROI) was defined as those voxels exhibiting both BOLD and ASL activation. Subjects were excluded from further analysis if fewer than 50 voxels met these criteria, resulting in the exclusion of three subjects from further study. Following ROI selection, data from the first visual task was excluded from further analysis.

### Voxel Scale BCP Analysis

To determine whether BCP estimation could be used to improve the precision of a dynamic CBF time series, BCP analysis was conducted on the measured ASL and BOLD time series obtained from the second visual task on each voxel within the previously defined ROI. Before performing the BCP analysis, known sources of physiological noise and linear scanner drifts were regressed out of the measured CBF- and BOLD-weighted time series [15], [16]. For each voxel, estimates of *f_0_* and *b_0_* were obtained by averaging the first 20 time points of the measured ASL and BOLD signals. The relative noise weighting terms 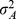 and 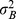 were estimated for each subject by calculating the mean variance of the measured ASL and BOLD signals (after physiological noise regression) in voxels with a CSF partial volume greater than 95% as estimated by auto-segmentation of the high resolution anatomical image with FSL’s FAST image segmentation tool [32]. The value of *k* that minimized Equation 4 was determined using a Golden-Section search algorithm [33]. This algorithm is initialized by bracketing the expected function minimum between two assumed values (e.g. *a* and *b*). Here we assumed that (corresponding to approximately for and) in order to minimize any *a priori* assumptions about its value. A third point (point *c*) is then chosen that is intermediate to the bracketing values, forming a triplet of test solutions (two brackets and an intermediate value). The minimum of a function is then found by evaluating it at a fourth point (point *d*) located 38.197% of the distance between the intermediate point and the more distant bracket (a fractional distance called the golden section). If the value of the function at *d* is lower than at *c*, then *c* becomes a new bracket point and *d* becomes the new intermediate value. If the value of the function is greater at *d* than at *c*, then *d* becomes the new bracket and *c* remains intermediate. In this way the distance between the brackets is reduced until a specified tolerance is reached. Here the tolerance was set to 0.001. At each test point, Equation 4 was evaluated by mapping each pair of measurements 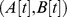 to the closest point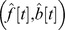 on a line defined by Equation 3 and the current value of 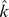.

To test the effect of voxel-wise BCP estimation, we examined signal-to-noise improvements by measuring the correlation (*r* ^2^) of the BCP-estimated CBF signal 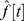 for each voxel in the active ROI with the stimulus-related regressor used in the GLM analysis and comparing it to the correlations of 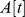 and 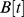 with the same regressor. In addition, we evaluated the precisions of 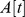 and 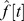 by calculating the standard deviation of each signal during the last 10 seconds of each stimulus, a time period usually assumed to represent a steady state of elevated CBF, and in the period 12.5–22.5 seconds after the cessation of each stimulus, a period of time during the BOLD post-stimulus undershoot. We also assessed whether BCP estimation introduced any bias in the estimate 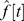 by comparing the ROI-averaged values of 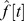 and 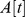 during the steady state period of activity and during the BOLD post-stimulus undershoot period. Because the hypercapnia calibration experiment used lacks sufficient precision to estimate *M* at the single voxel scale, we did not attempt to estimate λ for individual voxels.

### ROI-scale Analysis

BCP analysis was also conducted at the spatial scale of a region of interest in order to determine the feasibility of estimating the CMRO_2_-CBF coupling parameter λ with this technique. As described above, estimation of λ is feasible only if *M* and α_v_ are known. For this study we used the literature value of α_v = _0.2 for the CBF-venous CBV coupling constant [22] and an additional calibration experiment to make an ROI-scale estimate of *M* for each subject. To determine *M*, data from the two hypercapnia runs was first corrected for fluctuations due to physiological noise and linear drifts on a voxel-wise basis using a general linear model. Time series were then averaged across each subject’s ROI and across the two experiments to produce a single pair of CBF and BOLD time courses for each subject. Estimates of the baseline CBF and BOLD signals were obtained from these time courses by averaging the first 40 data points in the image series. Estimates of the steady-state response to hypercapnia were obtained by averaging the last 40 time points recorded while CO_2_ was being administered. The scaling parameter *M* was then calculated for each subject using the equation
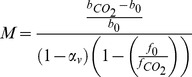
(5)where 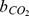 and 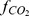 represent the steady state BOLD and CBF responses to hypercapnia, α_v_ is assumed to be 0.2, and λ is assumed to be zero.

Once values of *M* were obtained for each subject, the measured ASL and BOLD signals from the second visual task were corrected for fluctuations due to physiological noise and scanner drifts and then averaged-spatially across each subject’s ROI. The value of *k* for the average time courses was then estimated for a window the length of the entire functional run and λ was estimated using the formula 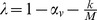. For comparison, λ was also estimated by a method more commonly used in calibrated BOLD studies. To obtain this reference estimate of λ, the measured CBF and BOLD responses of each subject were averaged over the last 10 seconds of each visual task to produce the average steady-state CBF and BOLD estimates *f_vt_* and *b_vt_*. These estimates were then plugged into the equation
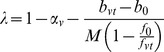
(6)


To investigate the possibility that λ or α_v_ fluctuate through the stimulus cycle, we next divided each subject’s ROI-averaged time series into four epochs: a transient active period (first 10 sec. of each stimulus), a steady state active period (last 10 sec. of each stimulus), a transient inactive period (first 10 sec after each stimulus) and a BOLD post-stimulus undershoot period (12.5–22.5 sec after the cessation of each stimulus). We then concatenated the data points corresponding to each of these four epochs, forming four BOLD/CBF time series pairs (per subject), each containing 16 data points from within a single epoch. We then used BCP analysis to estimate 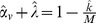 separately for each time series pair, under the assumption that systematic changes in λ and/or α_v_ would produce systematic differences in their sum from epoch to epoch.

## Results

### Voxel Scale BCP Analysis

**Figure 2:**
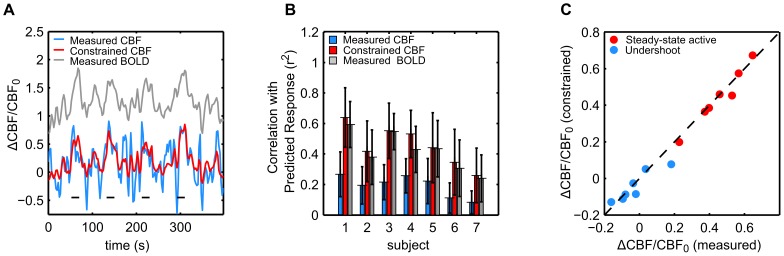
BCP estimation improves precision of CBF estimates without inducing estimation bias. **A**) Representative CBF time series from a single voxel within the visual cortex before (blue) and after (red) BCP estimation. For comparison, a scaled and shifted version of the measured BOLD signal is displayed in gray. Black lines indicate when the stimulus (8 Hz flashing checkerboard) was on. **B**) BCP significantly reduces the influence of noise on the CBF signal as measured by correlation with a predicted hemodynamic response. Height of the blue bars indicates mean correlation (r^2^) for each subject between measured CBF time series (after removal of known sources of physiologic noise) and a predicted CBF time course based on the convolution of the stimulus paradigm with a hemodynamic response function. Height of the red bars indicates mean correlation between BCP estimated CBF time series and the same predicted time course. For comparison, grey bars indicate the correlation between the measured BOLD response (after removal of known sources of physiologic noise) and the predicted time series. Error bars indicate the standard deviations of the calculated r^2^ values across the ROI. **C**) BCP estimation produces no detectable bias in CBF estimates of steady-state activation response or post-stimulus undershoot response. Scatterplot shows mean CBF responses for each subject during steady-state activation (red) and undershoot (blue) before (horizontal axis) and after (vertical axis) BCP analysis. No significant difference was observed between pre- and post-BCP estimates. *BCP: BOLD Constrained Perfusion. CBF: Cerebral Blood Flow. ROI: Region of Interest.*

Application of BCP estimation at the single voxel scale increased the correlation the CBF signal with the stimulus model and increased the precision of our estimates of CBF changes during steady-state active and BOLD-undershoot periods without introducing any apparent bias. Figure 2a displays a representative CBF time course from a single voxel after correction for known sources of physiological noise (blue) and after constraint by BCP analysis (red). For comparison, a scaled and shifted representation of the measured BOLD signal is also shown (gray). Black lines indicate when the visual stimulus was on. Note that the shape of the constrained CBF signal is similar, though not identical, to the BOLD signal, but that proper CBF scaling is maintained at the stimulus peaks. It is also interesting to note that many of the very large fluctuations in the measured CBF signal that occur between peaks are also represented (albeit in a less dramatic way) in the BOLD signal, and are thus attenuated but not eliminated from the constrained CBF signal. Figure 2b displays the mean correlation (*r* ^2^) of single voxel time series from within an ROI with a stimulus-related regressor. The height of the blue bars represents the mean *r* ^2^ of the measured CBF signal after correction for known physiological noise for each subject. The height of the red bars represents the mean *r* ^2^ of the BCP estimated CBF signal. For comparison, the grey bar represents the mean *r* ^2^ of the measured BOLD signal after correction for known physiological noise. Across subjects, the mean correlation of the BCP estimated CBF signal with the stimulus related regressor at the single voxel scale was 0.45+/−0.13 (mean +/− std.). This was significantly higher than the mean correlation of the measured CBF signal (0.19+/−0.07, *p*<0.01, pairwise t-test) and the measured BOLD signal (0.42+/−0.12, *p* = 0.026, pairwise t-test).

In addition to increasing the correlation of the CBF signal with the stimulus model, BCP estimation increased the precision of estimated changes in CBF during both the steady-state active and BOLD post-stimulus undershoot periods. Across subjects, during the active period, the mean standard deviation of the single voxel CBF signal as a fraction of the baseline was 0.38+/−0.14 for the measured signal and 0.22+/−0.08 for the BCP-estimated signal (*p<0.01* pairwise t-test). Similarly, during the undershoot period, the mean standard deviation of the single voxel CBF signal was 0.38+/−0.12 for the measured CBF signal and 0.14+/−0.04 for the BCP-estimated signal (*p<0.01* pairwise t-test). For comparison, the mean value of 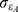
was 36+/−15% of the baseline ASL signal or 28+/−5 signal units. The mean value of 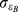
was 0.5% +/−0.2% of the baseline BOLD signal or 56+/−18 signal units across subjects. No bias was observed in the BCP estimated CBF signals during either the steady-state active period or the post-stimulus undershoot period. Figure 2c displays the mean change in CBF as a fraction of baseline CBF signal for each subject during the active (red) and undershoot (blue) periods for the measured (horizontal axis) and BCP estimated (vertical axis) CBF signals. Across subjects the mean change in CBF during the steady-state active period was 0.46+/−0.14 (mean +/− std.) for the measured CBF signal and 0.44+/−0.15 for the BCP estimated CBF signal (*p* = 0.3, pairwise t-test). The mean change in CBF during the undershoot period was −0.02+/−0.11 for the measured CBF signal and −0.04+/−0.08 for the BCP estimated CBF signal (*p = *0.31, pairwise t-test). For the measured BOLD signal, the mean change across subjects was 0.014+/−0.004 (mean +/− std.) during the steady-state active period and −0.002+/−0.004 (mean +/− std.) during the undershoot period.

### ROI-scale Analysis

**Figure 3:**
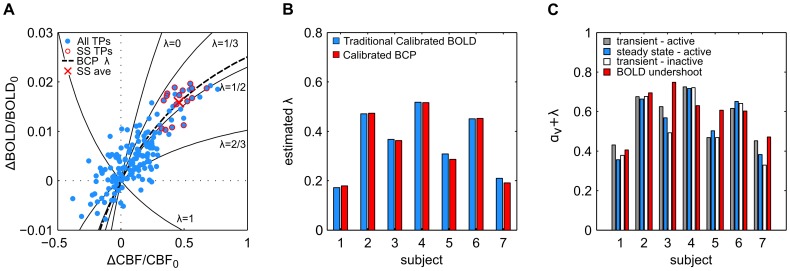
Calibrated BCP allows estimation of CMRO_2_-CBF coupling without prior knowledge of the stimulus paradigm. **A**) Representative, ROI-averaged single subject (subject 2) data comparing traditional and BCP approach to estimating λ, the ratio of changes in CMRO_2_ to changes in CBF evoked by a stimulus. In traditional calibrated BOLD analysis, BOLD and CBF measurements collected at time points (TPs) during which the stimulus response is assumed to be in a steady state (SS). These measurements (red circled dots) are averaged into a single measurement (red ‘X’). The location of the ‘X’ in the BOLD-CBF plane determines the coupling ratio λ. Conversely, calibrated BCP estimation requires no knowledge of the stimulus pattern. All data points within a time window (here, the length of the experiment) are fit to a cost function (Equation 4 in text) using a mathematical model (here Equation 3 in text) to link BOLD and CBF fluctuations. The value of λ that minimizes the difference between the measured and estimated BOLD and CBF values given the relative noise (dashed black line) determines the coupling ratio. **B**) Estimates of λ produced by blind Calibrated BCP analysis agree with those produced by traditional calibrated BOLD analysis. Height of blue bars indicates traditional calibrated BOLD estimate for each subject. Height of red bars indicates the calibrated BCP estimate for a window the length of the full time series. No significant difference between the two was detected. **C**) Epoch-based BCP analysis does not reveal evidence of systematic variation of model parameters with stimulus cycle. Height of bars indicates the estimated sum of the model parameters α_v_ and λ during the transient active (gray), steady state active (blue), transient inactive (white), and BOLD undershoot periods (red). Considerable differences between steady state and undershoot estimates may be seen in several subjects; however, no systematic differences were detectable across the group. *BCP: BOLD Constrained Perfusion. ROI: Region of Interest. CMRO_2_: Cerebral Metabolic Rate of Oxygen. CBF: Cerebral Blood Flow.*

BCP estimation at the ROI level yielded estimates of λ in good agreement with those produced by traditional calibrated BOLD techniques despite the blind application of the BCP estimation method to the entire time series. Figure 3 displays the estimates of λ found for each subject by traditional analysis and by BCP analysis using the heuristic model. Figure 3a displays results from a single subject (subject 2), demonstrating the difference between estimating λ by traditional calibrated BOLD methods and by BCP estimation. In traditional calibrated BOLD analysis, BOLD and CBF measurements collected during a period of steady-state activity (red circled dots) are averaged together to produce a single estimate of the change in BOLD and CBF signal associated with the stimulus (red ‘X’). The location of this point in the CBF-BOLD plane determines the value of 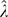. In contrast, with BCP analysis, all (CBF, BOLD) data points from within a chosen time-window, in this case the length of the entire functional run, are fit to a BOLD-CBF relationship defined by a BOLD signal model. The value of 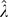 that minimizes Equation 4 determines the CMRO_2_-CBF coupling ratio. Figure 3b displays the value of 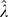 estimated for each subject using the BCP or traditional approach. Across subjects the mean value of 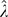 estimated by BCP analysis of the whole time series was 0.35+/−0.14 (mean +/− std.) and by traditional calibrated BOLD, 0.36+/−0.13 (*p = *0.26, paired t-test). Across subjects the mean scaling parameter *M* was found to be 0.11+/−0.04.

As is shown in Figure 3c, there were not any clear systematic differences across subjects in the values of λ+ α_v_ estimated at different stages of the stimulus cycle. Across subjects, 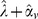 was estimated to be 0.57+/−0.12 during the transient active period, 0.55+/−0.14 during the steady-state period, 0.53+/−0.15 during the transient off period, and 0.59+/−0.12 during the post stimulus undershoot. Not one epoch was found to produce an estimate of α_v_+λ that was significantly different from another across subjects (p>0.2 for all pairwise t-tests, even without correction for multiple comparisons).

## Discussion

In this study we report a new method of measuring dynamic CBF fluctuations by combining information obtained through simultaneous acquisition of ASL and BOLD image time series. This approach takes advantage of the favorable features of both time series. The ASL measurement is directly proportional to CBF, but the low signal to noise ratio makes it difficult to assess dynamics. The BOLD signal has much better sensitivity, but is related to the underlying CBF fluctuations in a complicated and nonlinear way. To simplify this relationship, we incorporated a recent model of the BOLD effect. The BOLD and ASL signals are then essentially treated as two independent but noisy windows into the same underlying physiological process, so that by constraining the CBF fluctuations to be consistent with the BOLD signal model, we may substantially decrease the influence of noise on the CBF time series and increase the precision of CBF estimates. Importantly, the BOLD constrained perfusion (BCP) estimation procedure does not require any prior knowledge of the stimulus, suggesting that the method may be applicable to complex tasks in addition to conventional block and event-related stimulus designs.

### Reducing the Influence of Noise on CBF Estimates and Time Series Measurements

To test the method we used data from a simple, block-design visual task for which we believed we could generate a fairly accurate, *a priori* model of dynamic CBF fluctuations. We then compared the correlation of measured and BCP-estimated CBF time series with the predicted model as a metric for the improvement in SNR. We found that the value of the BCP estimated CBF signal was on average more than 200% that of the measured CBF signal based on ASL alone at the single voxel scale and that it was comparable to, and even slightly greater than, the *r* ^2^ value of the measured BOLD signal, suggesting a substantial decrease in the influence of noise on the CBF time series. We noted that the shape of the constrained CBF time series was similar, though not identical, to that of the measured BOLD time series. They are not identical because both the BOLD and CBF signals are assumed to contain some noise, which differentiates BCP estimation from a simple, non-linear regression analysis. However, it is not surprising that the estimated CBF is similar to the measured BOLD, given the higher SNR of the BOLD signal. In addition, we noted that, interestingly, many of the very large, inter-stimulus fluctuations in the measured CBF signal were also present, though to a lesser extent, in the BOLD signal, and were thus reduced in magnitude but not absent in the constrained CBF signal. We cannot conclude definitively whether these correlated fluctuations partly represent real fluctuations in CBF (perhaps related to opening and closing of eyes between stimuli or “resting state” activity) or whether they represent correlated noise in the BOLD and CBF signals. Several features of our image acquisition and processing protocol, however, reduce the likelihood that these fluctuations are pure artifacts. First, the CBF and BOLD signals are acquired from separate spiral readouts, making it unlikely that random readout noise or k-space spikes would be correlated across the two signals. Second, the surround subtraction procedure used to produce the CBF signal should further reduce the correlation of its noise with that of the BOLD signal. For example, while rapid fluctuations in the static tissue signal that are precisely timed with control images will produce correlated fluctuations in the BOLD and CBF signals, those that are precisely timed with tag images will produce anti-correlated fluctuations, and those that last longer than a tag-control triplet will be preserved in the BOLD but eliminated from the CBF signal. Still, further work must be done to investigate the source of these correlated inter-stimulus fluctuations, and if they prove to be artifacts, to account for them.

We further investigated the effect of BCP analysis on the precision of the CBF signal by measuring the standard deviation of CBF measurements taken during a period of presumably steady-state activity and during the post-stimulus BOLD undershoot. Again, we found that BCP analysis significantly reduced the influence of noise on the measurements, reducing the standard deviation of the measurements by approximately 40% during the active period and by approximately 60% during the undershoot period.

We were concerned that despite the improvement in the precision of our measurement, there might be some bias in the magnitude of the BCP estimated CBF fluctuations as compared to the measured ASL signal, which we assume, on average, reflects the true magnitude of CBF fluctuations. We were particularly concerned about this possibility because we assume in applying the BCP estimation approach that the parameters of our BOLD signal model are fixed throughout the duration of a chosen time window, which in our analysis encompassed the entire experiment. As discussed above, despite the simplicity of our experimental design, the stimulus we chose consisted of several epochs (e.g. rest, activation, post-stimulus BOLD undershoot, and transitions between activity and rest) during which several of our BOLD model parameters (in particular λ and possibly α_v_ as well) might be expected to change significantly, and we anticipated that the non-stationarity of these parameters might bias the BCP estimate of the CBF signal.

To determine whether bias was significant in the BCP estimated signal, we averaged CBF measurements taken during the steady-state period of activity and during the post-stimulus undershoot period and compared them to average BCP estimates of the same periods. We found that during the steady-state activity period, the average BCP estimate was only 1.6% of the baseline signal lower than the average measurement and that during the undershoot period the average BCP estimate was only 2.1% lower. Neither of these differences was statistically significant. While we cannot conclude definitively from this finding that the BCP estimate is unbiased or that this finding is applicable to all stimulus paradigms, we take it as an encouraging sign that the BCP CBF estimate is reasonably robust despite the potential weakness of the parameter stationarity assumption. Further experiments will be important to test this potential limitation of the method.

### Estimating CMRO_2_-CBF Coupling

BCP estimation with the heuristic model yields a set of CBF estimates as well as an additional parameter estimate, 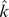. We noted that the value of this parameter alone could not be interpreted in a physiological sense. However, we hypothesized that if values for the CBF-CBV coupling parameter, α_v_, and the BOLD model scaling factor, *M*, could be obtained, then the value of 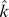 could be used to calculate an estimate of the ratio of fluctuations in CMRO_2_ to CBF, 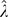, throughout the analysis window. Because of the imprecision of the calibration experiment required to estimate *M*, the test of this hypothesis was conducted at the scale of a region of interest in the visual cortex. To determine whether the blind application of BCP analysis to the complete time series could produce an accurate estimate of λ, we compared estimates of λ produced by a traditional calibrated BOLD technique to estimates produced by BCP analysis of the complete time series. We found that λ estimates were highly consistent between the traditional and BCP estimation techniques, with no significant differences between the two. We then divided the time series into distinct epochs in order to look for systematic differences in the sum of λ and α_v_ during the steady state active period, the post-stimulus BOLD undershoot period, and the transition periods as the stimulus was turned on and off. We looked at the sum of λ and α_v,_ rather than λ alone, because in transition periods we cannot be certain that α_v_ maintains its steady state value. Across subjects, no systematic differences in the sum 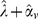 were found between any epoch pairs. Again, these findings are encouraging, as they suggest that potentially divergent CBF and BOLD dynamic transients are not having a strong biasing effect on our estimates of *k*; however, more work will be required to determine conclusively whether the apparent lack of systematic difference is attributable to an underlying physiological process or simply to signal noise.

### The BOLD Post-stimulus Undershoot

The lack of evidence of systematic bias in the BCP estimates of CBF and *k* during the BOLD undershoot period is somewhat surprising given our current understanding of its etiology. The origin of the BOLD post-stimulus undershoot has been a topic of considerable debate for nearly two decades. Several studies have found the undershoot to be consistent with a slow return to baseline of CMRO_2_ compared with CBF [34], [35], while others have found it to be consistent with a slow return of venous blood volume [36], [37] or a post-stimulus CBF undershoot [37]. Transient uncoupling of CBF and CMRO_2_ dynamics would result in changes in λ (increased for a slow CMRO_2_ recovery and decreased for a CBF undershoot at baseline CMRO_2_). The model for the BOLD signal used in the BCP analysis does not include the possibility of a slow return of blood volume explicitly, so we would expect this effect to appear as a slow recovery of CMRO_2_ and a correspondingly higher value of λ (i.e., the basic problem is that these two potential effects can produce similar BOLD responses). Each of these potential undershoot mechanisms suggest that *k* (or λ+α_v_) should be significantly different in the active and undershoot states, and that as a result, our estimate of CBF in the undershoot period should be systematically biased if we blindly apply BCP to a long time series. The reason we do not see this bias may be because the BOLD and CBF fluctuations in the undershoot period are relatively small. As Figure 3a demonstrates, CBF-BOLD contours representing distinct values of λ converge at the origin. As a result, near the origin small deviations in the CBF-BOLD plane produce large changes in λ. Thus in this regime, systematic errors in the estimated CBF signal due to a biased estimate of *k* are likely to be small, especially compared to the random error due to noise. This has both positive and negative implications for BCP analysis. On the positive side, it suggests that even large changes in λ during an undershoot should not cause dramatic bias in the CBF estimates made in that period, as we have seen here. On the negative side, it suggests that as the magnitude of CBF and BOLD fluctuations within a window of interest decrease, the precision of BCP estimates of λ should decrease as well.

### Potential Applications for BCP Analysis

The two principal findings of this work were (1) that the blind application of BCP-analysis to voxel scale CBF time series increased their correlation with the hemodynamic model and increased the precision of CBF estimates both in periods of steady-state activity and post-stimulus undershoot without producing significant estimation bias, and (2) that the blind application of BCP-analysis to ROI-scale BOLD and CBF data produced an estimate of the CMRO_2_-CBF coupling parameter λ that was highly consistent with one produced by traditional, steady-state calibrated BOLD analysis. These findings are encouraging, as they suggest that transient fluctuations in our BOLD model parameters (λ and α_v_) may not dramatically bias our estimates of instantaneous CBF or CMRO_2_-CBF coupling over a window of time if the underlying hemodynamic and metabolic activity is coupled in a relatively stationary way, as was the case in these experiments. The findings presented here suggest that BCP analysis may be immediately useful in the study of the hemodynamic responses of small regions of interest or even single voxels to simple block-design stimuli, as a way of improving the precision of CBF estimates. Similarly, BCP has the potential to be useful in the study of stimuli that cannot be presented repeatedly or for prolonged periods, either because they are noxious or produce habituation or sensitization. BCP analysis could also be potentially applied to the calibration of the BOLD response by fitting for the parameter *k* during a CO_2_ challenge and calculating *M* based on the assumption that λ = 0; however, given the CO_2_ challenge often lasts several minutes, BCP may not produce a more precise estimate of *M* than is achievable with simple temporal averaging.

Looking forward, we hope that BCP analysis will prove to be a useful tool in the quantitative study of hemodynamic and metabolic activity associated with more natural neural tasks, such as watching movies, listening to music, or even rest, tasks that are difficult to study with traditional calibrated BOLD techniques because the temporal pattern of CBF and BOLD fluctuations may not be predicable or replicable with repeated stimuli. To date, neural tasks of this type have typically been studied in a qualitative or semi-quantitative manner. Several groups [38], [39] have used BOLD imaging alone to investigate the patterns of neural activity associated with watching popular films and found significantly correlated signal fluctuations not just across regions within a single brain, but across the brains of multiple subjects, suggesting that such natural stimuli might be used to drive blood flow and oxygen metabolism fluctuations throughout the brain, allowing many regions to be studied at once. Similarly, resting state BOLD fMRI has been used extensively to map the spatial and temporal patterns of hemodynamic activity that occur when a subject lies quietly in the MR scanner [40]–[42]. ASL has also been used for this purpose [43], [44] and the two modalities have even been combined in a semi-quantitative fashion to demonstrate that the ratio of BOLD fluctuations to ASL fluctuations at rest is closer to the ratio associated with a visual task than an iso-metabolic breathing task, suggesting a metabolic basis for resting state BOLD fluctuations [45]. The consensus produced by this body of work is that hemodynamic and metabolic activity in the brain is highly coordinated even in nominal states of rest. However the magnitude of this activity, and thus its importance in maintaining homeostasis, remains poorly understood. If BCP estimation may be applied to quantitatively measure the CBF and CMRO_2_ fluctuations associated with natural neural activity, it could provide important insights into the physiology of complex neural processing and how it is altered by disease.

### Potential Limitations and Future Work

Despite the promising results of this proof-of-principle experiment, we acknowledge that the results presented here do not demonstrate conclusively that BCP analysis will prove to be robust under more general experimental conditions, and more study will be necessary before BCP analysis is ready to be used to study the physiology of complex neural processing. A key issue that requires further study is the sensitivity of the BCP estimation approach to the dynamics of the BOLD signal. As discussed above, the BOLD response to neural activity is notable for several transient features that have been observed in various studies including initial dips [26], early overshoots [25], and post-stimulus undershoots [24]. While no definitive dynamic BOLD model has yet been described, both experimental [35], [46] and theoretical [27], [36], [47] analyses agree that these transient features occur due to differences in the dynamic responses of CMRO_2_, CBF, CBV to neural stimuli. This poses a potential challenge for BCP estimation, as the simplifications made to the BOLD signal model in order to reduce it to a function of CBF and a few unknown parameters implicitly requires the assumption that these physiological variables are dynamically synchronized, at least over the finite length of a window of time and within the temporal resolution of our measurements. In this study we looked for evidence of bias due to this assumption by comparing the mean responses of measured and BCP estimated CBF time series both in the active state and during the post-stimulus undershoot, by comparing estimates of λ produced by blind BCP estimation with those made by traditional calibrated BOLD analysis, and by comparing BCP estimates of the sum λ+α_v_ at different stages of the stimulus cycle. None of these tests revealed evidence of bias, even during the period of the BOLD post-stimulus undershoot. This is quite encouraging, however, the lack of evidence of bias in this study cannot be taken as definitive proof that BCP analysis is robust to these transients, nor does it guarantee that BCP analysis will be robust to transient dynamics under more general experimental conditions. To test this assumption more rigorously, we are currently working to develop visual stimuli that continuously drive CBF and CMRO_2_ in ways that will allow us to carefully examine how both how the dynamics and amplitudes of CBF and BOLD fluctuations influence the accuracy and precision of BCP estimation. A useful tool in assessing the robustness of BCP analysis under these more general conditions may be ASL with background suppression. Several methods of acquiring background suppressed ASL images have recently been developed [48]–[50], though they share the common strategy of reducing noise from the static tissue compartment through the application of multiple inversion pulses timed to null the static tissue signal at the time of image acquisition [51]. An advantage of ASL with background suppression is that it achieves SNR gains independently of the BOLD effect, which makes it less vulnerable to the sources of potential bias in BCP analysis. CBF time series produced by background suppressed ASL may thus prove to be useful reference functions for determining BCP estimation bias in future studies.

### Conclusions

We have presented here a proof-of-principle demonstration of the feasibility of improving the precision of dynamic estimates of CBF by combining information from simultaneously acquired ASL and BOLD images through a technique we term BOLD Constrained Perfusion (BCP) estimation. Further, we have shown that, under the condition that a calibration experiment is conducted, the BCP approach may be utilized to obtain quantitative information about the coupling of CMRO_2_ and CBF fluctuations. Importantly, we have demonstrated that this technique may be used without taking into consideration the temporal dynamics of the stimulus presented, suggesting that it may be useful in the quantitative study of hemodynamic and metabolic responses to neural tasks that cannot be easily modeled temporally. Further studies are required to investigate and if necessary correct for the sensitivity of the BCP approach to the dynamics of CMRO_2_, CBF, and CBV; however, the results presented in this initial test are quite promising and suggest that, despite its simplicity, BCP analysis may improve our ability to estimate CBF and CMRO_2_ fluctuations under conditions that are currently challenging to study with traditional calibrated BOLD techniques.

## Supporting Information

Figure S1 **Calibrated BCP Estimation with the Davis model.** In this bar chart, the height of blue bars indicates traditional calibrated BOLD estimate of λ, the ratio of evoked changes in CMRO_2_ to CBF, for each subject. The height of red bars indicates the BCP estimate. Dark colored bars represent estimates based on the Davis model. Light Colored bars represent estimates based on the heuristic model. No significant differences between BCP and traditional estimates produced by the same model were observed. However, a small but significant difference in the estimates produced by the two models was observed, regardless of whether BCP or traditional calibrated BOLD estimation was used. *BCP: BOLD Constrained Perfusion. ROI: Region of Interest. CMRO_2_: Cerebral Metabolic Rate of Oxygen. CBF: Cerebral Blood Flow.*
(TIFF)

Figure S2 **Danger of attributing physiological significance to simultaneously estimated values of λ and M.** In the heuristic model (Equation 3 in Text), The CMRO_2_-CBF coupling parameter, λ, and the scaling parameter, M, may be lumped into a single parameter, *k*, when both of their values are unknown. BCP analysis may then still be used to improve CBF estimates, although *k* has no real physiological meaning. In the Davis model (Equation S1 in Document S1), λ and M cannot be lumped together and must be estimated simultaneously from the data if both are unknown. However, if estimated in this manner, their values will still not be interpretable physiologically because the BOLD-CBF relationship is not uniquely defined. The plot above illustrates this point, displaying two nearly identical BOLD-CBF relationships defined by the Davis model for two very different pairs of λ and M. *BCP: BOLD Constrained Perfusion. CMRO_2_: Cerebral Metabolic Rate of Oxygen. CBF: Cerebral Blood Flow.*
(TIFF)

Document S1 **Discussion of BCP Analysis with Davis model.** In theory BCP estimation should be applicable to a variety of mathematical models of the BOLD signal. Here we repeated our analysis using the Davis model (Equation S1 in Document S1) instead of the Heuristic model (Equation 3 in the Text) to constrain the relationship between BOLD and CBF measurements.
(DOC)
