# Supplementary figures and images for: A Novel Method of Combining Blood Oxygenation and Blood Flow Sensitive Magnetic Resonance Imaging Techniques to Measure the Cerebral Blood Flow and Oxygen Metabolism Responses to an Unknown Neural Stimulus
Source: PLoS One. 2013 Jan 31;8(1):e54816. doi: 10.1371/journal.pone.0054816 (PMC3561406; doi:10.1371/journal.pone.0054816)

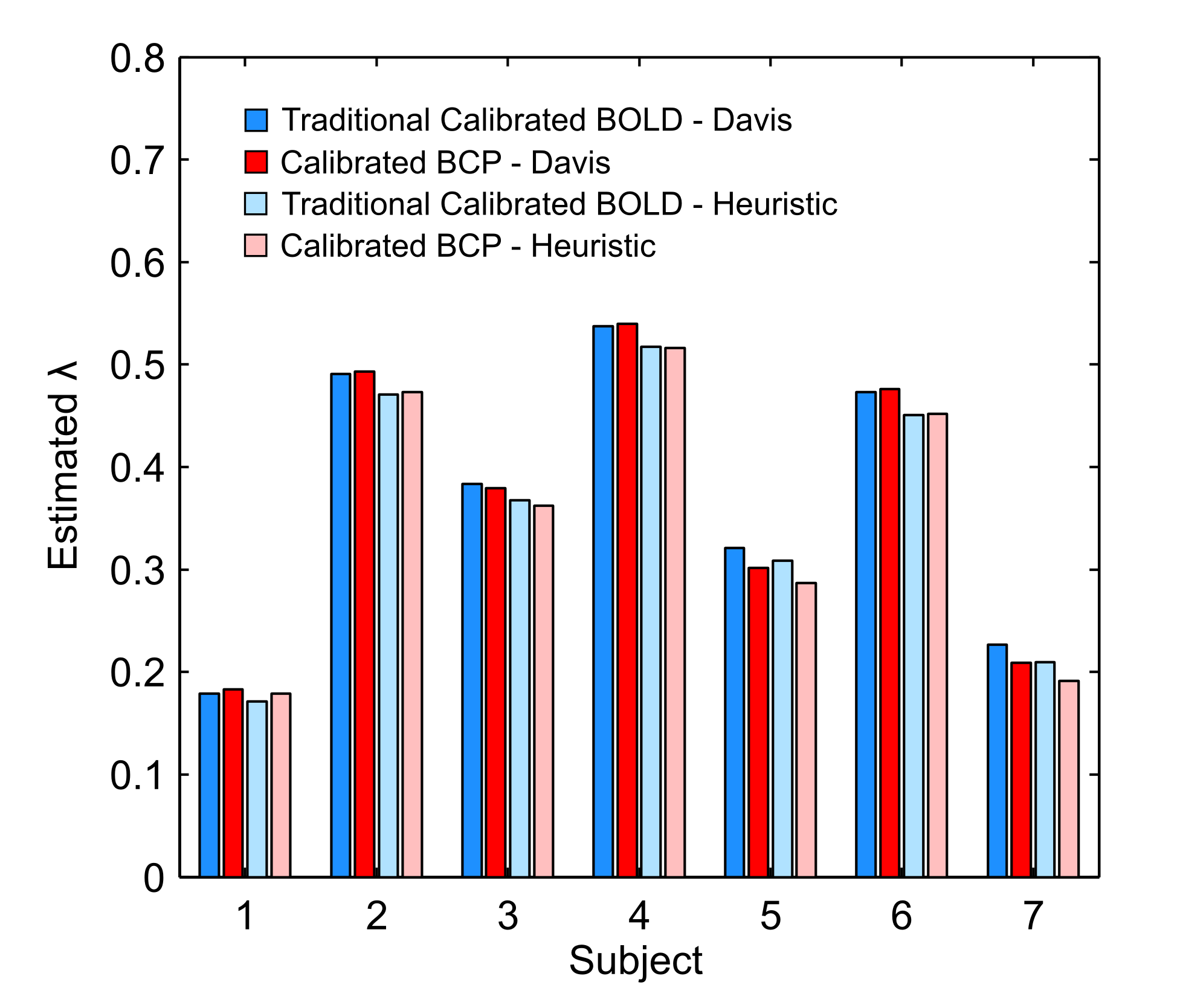

Supplement: Figure S1 — Calibrated BCP Estimation with the Davis model. In this bar chart, the height of blue bars indicates traditional calibrated BOLD estimate of λ, the ratio of evoked changes in CMRO2 to CBF, for each subject. The height of red bars indicates the BCP estimate. Dark colored bars represent estimates based on the Davis model. Light Colored bars represent estimates based on the heuristic model. No significant differences between BCP and traditional estimates produced by the same model were observed. However, a small but significant difference in the estimates produced by the two models was observed, regardless of whether BCP or traditional calibrated BOLD estimation was used. BCP: BOLD Constrained Perfusion. ROI: Region of Interest. CMRO2: Cerebral Metabolic Rate of Oxygen. CBF: Cerebral Blood Flow. (TIFF) [file pone.0054816.s001.tiff]

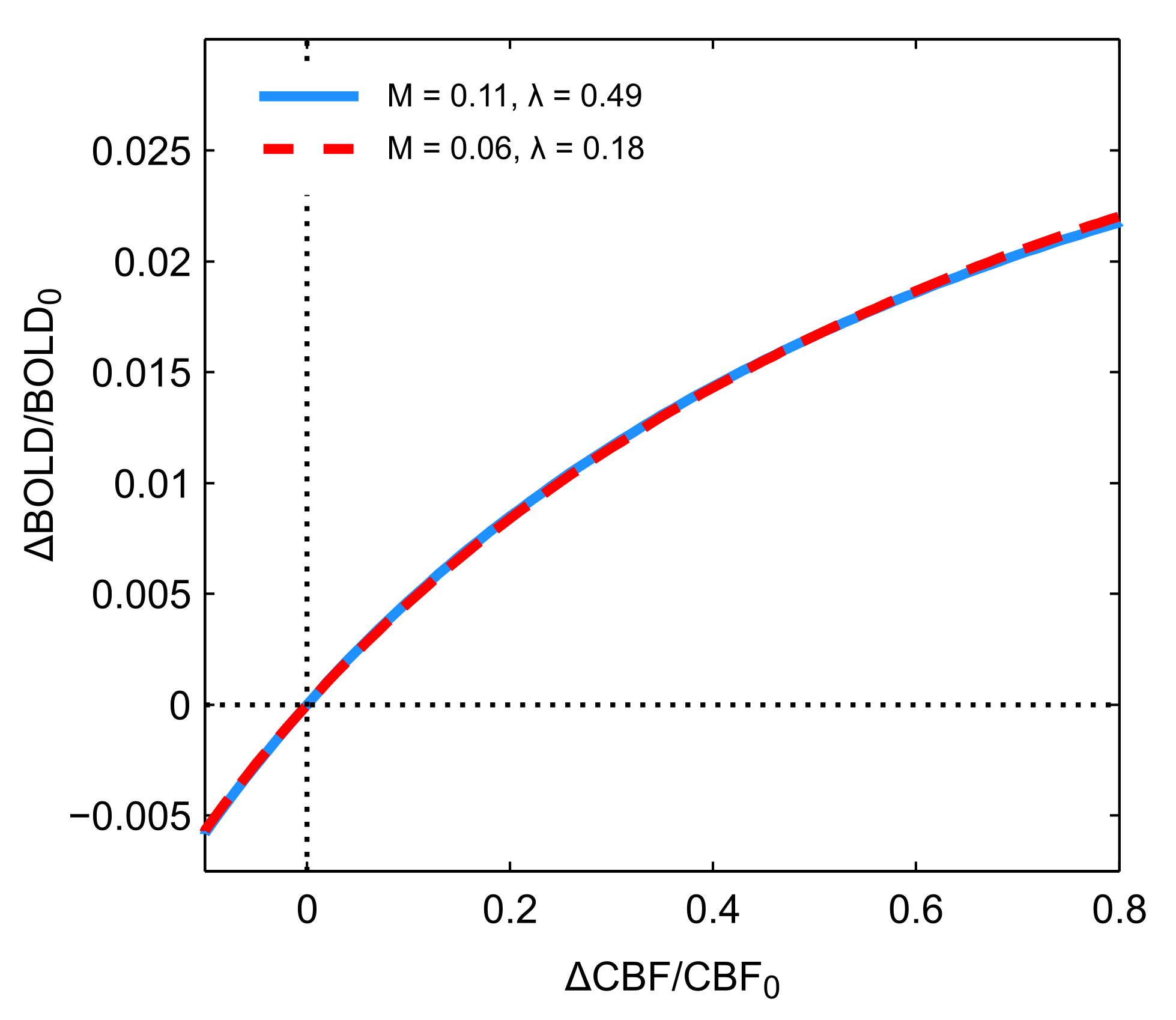

Supplement: Figure S2 — Danger of attributing physiological significance to simultaneously estimated values of λ and M. In the heuristic model (Equation 3 in Text), The CMRO2-CBF coupling parameter, λ, and the scaling parameter, M, may be lumped into a single parameter, k, when both of their values are unknown. BCP analysis may then still be used to improve CBF estimates, although k has no real physiological meaning. In the Davis model (Equation S1 in Document S1), λ and M cannot be lumped together and must be estimated simultaneously from the data if both are unknown. However, if estimated in this manner, their values will still not be interpretable physiologically because the BOLD-CBF relationship is not uniquely defined. The plot above illustrates this point, displaying two nearly identical BOLD-CBF relationships defined by the Davis model for two very different pairs of λ and M. BCP: BOLD Constrained Perfusion. CMRO2: Cerebral Metabolic Rate of Oxygen. CBF: Cerebral Blood Flow. (TIFF) [file pone.0054816.s002.tiff]
