# Supplementary material for: A Novel Method of Combining Blood Oxygenation and Blood Flow Sensitive Magnetic Resonance Imaging Techniques to Measure the Cerebral Blood Flow and Oxygen Metabolism Responses to an Unknown Neural Stimulus
Source: PLoS One. 2013 Jan 31;8(1):e54816. doi: 10.1371/journal.pone.0054816 (PMC3561406; doi:10.1371/journal.pone.0054816)
Supplement: Document S1 — Discussion of BCP Analysis with Davis model. In theory BCP estimation should be applicable to a variety of mathematical models of the BOLD signal. Here we repeated our analysis using the Davis model (Equation S1 in Document S1) instead of the Heuristic model (Equation 3 in the Text) to constrain the relationship between BOLD and CBF measurements. (DOC) [file pone.0054816.s003.doc]

**Supplemental Section**

*Application of the Davis model to BCP Estimation*

In theory, BCP estimation should be compatible with any BOLD signal model that can be expressed in terms of fluctuations in cerebral blood flow. The Davis model is one of the most commonly used BOLD signal models used today in calibrated BOLD experiments, and, as such, we verified that the Davis model could be applied to BCP estimation. The Davis model may be expressed as follows [1,2].

In addition to the BOLD and CBF terms, this model contains four additional parameters: *M*,
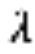
, and α_v_, which have the same significance as in the heuristic model, though the value of *M* for the two models will generally be different, and an additional parameter, β, that accounts for the differential effects of tissue water diffusion around large and small blood vessels on the apparent rate of signal decay $R_{2}^{*}$. Note that, because of the form of equation S1, these four parameters cannot be lumped together, as with the heuristic model, and thus must be fit simultaneously to the data or estimated by other means. Here we chose to use values from the literature to approximate α_v_ = 0.2 and β = 1.3 [3,4]. At the single voxel scale, both *M* and λ were fit simultaneously to the data, and at the ROI scale, *M* was estimated from a hypercapnia experiment while λ was fit to the data. At the single voxel scale, a different approach was required to minimize Equation 4 because both *M* and λ were unknown. To accomplish this, we used the MATLAB minimization algorithm *fminsearch* [5] with the additional constraints that the value of
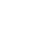
 *M* could not be smaller than the largest measured fractional change in the BOLD signal or larger than 0.5. *M = 0.1* and λ = 0.5 were used as starting points for the minimization. We compared the resulting time courses to those produced with the heuristic model by measuring the correlation ($r^{2}$) of each BCP estimated CBF time course produced by the Davis model with its counterpart produced by the heuristic model. The ROI scale analysis was performed in the same way as with the heuristic model with the following exceptions. First, Equation 4 was minimized using equation S1 as a constraint on $\hat{b}[t]$ rather than Equation 3. This required the direct optimization of $\hat{\lambda}$ rather than $\hat{k}$, which was initially bracketed within the domain $-20\leq\hat{\lambda}\leq20$. Second, the value of *M* was estimated from the hypercapnia experiment using the Davis model rather than the heuristic model

$$M=\frac{\frac{b_{CO_{2}}-b_{0}}{b_{0}}}{\left(1-\left(\frac{f_{CO_{2}}}{f_{0}}\right)^{\alpha_{v}-\beta}\right)}$$

Third, the traditional calibrated-BOLD estimate of $\lambda$to which each BCP estimate was compared was calculated from the Davis model rather than the heuristic model.

$$\hat{\lambda}=\frac{\left(1-\frac{b_{v}t-b_{0}}{M\bullet b_{0}}\right)^{\frac{1}{\beta}}-\left(\frac{f_{vt}}{f_{0}}\right)^{\frac{\alpha}{\beta}-1}}{\left(\frac{f_{vt}}{f_{0}}-1\right)\left(\frac{f_{vt}}{f_{0}}\right)^{\frac{\alpha}{\beta}-1}}$$

*Results*

The estimated CBF time courses generated by the Davis model analysis were highly similar to those generated with the heuristic model. Across all subjects and ROIs, the median inter-model $r^{2}$ value was 0.999. There were some individual voxel time courses for each subject that differed considerably; however, fewer than 15% of all voxels produced $r^{2}$ < 0.95 and only 10% produced $r^{2}$ < 0.9. At the ROI scale, as with the heuristic model, the values of λ obtained by BCP analysis with the Davis model agreed very well with those obtained by traditional calibrated BOLD analysis with the Davis model (Figure S1). Across subjects, the mean value of λ estimated by BCP analysis with the Davis model was 0.37 +/- 0.14. The mean value of
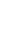
 λ estimated by traditional calibrated BOLD with the Davis model was also 0.37 +/- 0.14 (*p* = 0.32 vs. BCP estimate with Davis model, paired t-test). Both of these estimates were slightly ($\approx5\%$) but statistically significantly higher (p < 0.01) than their counterparts estimated with the heuristic model, suggesting that while choice of BOLD signal model may bias the BCP estimate, it does so in the same way that it biases estimates made by traditional calibrated-BOLD analysis.

*Discussion*

The primary analysis in this work was conducted using a simple heuristic model to link BOLD fluctuations to CBF (Equation 3). However, BCP estimation should, in theory, be able to accommodate any closed-form BOLD model. To examine the feasibility of using other BOLD models, we applied the Davis model (Equation *S1*) to the voxel scale estimation of CBF time series and the ROI scale estimation of the coupling parameter λ. At both spatial scales we found estimates produced by the Davis model to be in good agreement with those produced by the heuristic model. We did observe a small but significant difference between estimates of λ produced by the Davis model and the heuristic model at the ROI scale, indicating that model choice will affect the accuracy of the estimate; however, we observed this difference regardless of whether BCP or traditional calibrated BOLD methods were used to calculate λ, reflecting the fact that imperfect BOLD signal models will always limit the accuracy of CMRO_2_ estimates based on BOLD imaging and that this limitation is not particular to BCP estimation.

An advantageous feature of the heuristic model is that the biophysical parameters of the model (*M, α_v_,* and λ) may be lumped together into a single scaling parameter, *k*, making the minimization of Equation 4 fairly straightforward even when more than one of these parameters are unknown, as was the case for our voxel scale analysis. This is not possible with many BOLD signal models, including the Davis model, meaning that more than one parameter may have to be fit to the data in such cases. We note that in our voxel scale analysis with the Davis model, we simultaneously fit for the values of *M* and λ, producing estimates of both for each time course. However, while it might be tempting to interpret the values of these fit parameters physiologically, we caution against attributing any more physical significance to them than to the heuristic model parameter, *k*. We do so because the shape of the BOLD-CBF relationship described by the Davis model is not uniquely defined by a single set of parameters λ and *M*. Figure S2 illustrates this point, displaying nearly identical BOLD-CBF curves produced by two very different sets of λ and *M* values. The BOLD-CBF relationship is not uniquely defined because the magnitude of the BOLD response depends on both on neurovascular coupling and on the concentration of deoxyhemoglobin in the baseline state. Thus an independent measurement of *M* is necessary to produce a unique estimate of λ, regardless of the BOLD signal model used in the analysis.

**References**

1. Davis TL, Kwong KK, Weisskoff RM, Rosen BR (1998) Calibrated functional MRI: mapping the dynamics of oxidative metabolism. Proc Natl Acad Sci USA 95: 1834–1839.

2. Blockley NP, Griffeth VEM, Buxton RB (2012) A general analysis of calibrated BOLD methodology for measuring CMRO2 responses: Comparison of a new approach with existing methods. NeuroImage 60: 279–289. doi:10.1016/j.neuroimage.2011.11.081.

3. Chen JJ, Pike GB (2010) MRI measurement of the BOLD-specific flow–volume relationship during hypercapnia and hypocapnia in humans. NeuroImage 53: 383–391. doi:10.1016/j.neuroimage.2010.07.003.

4. Mark CI, Fisher JA, Pike GB (2010) Improved fMRI calibration: Precisely controlled hyperoxic versus hypercapnic stimuli. NeuroImage: 1–10. doi:10.1016/j.neuroimage.2010.08.070.

5. The MathWorks Inc. (2011) MATLAB.
